# Public health nurse reflections on implementing the New Families home visiting programme: A qualitative study

**DOI:** 10.1002/nop2.1996

**Published:** 2023-09-07

**Authors:** Bettina Holmberg Fagerlund, Kari Glavin

**Affiliations:** ^1^ Faculty of Health Sciences VID Specialized University Oslo Norway

**Keywords:** child, child health services, family, father, home visit, mother, parent, public health nursing

## Abstract

**Aim:**

To investigate reflections of public health nurses (PHNs) on implementing the New Families programme, a supplement to the usual Norwegian child health centre programme. It involves user‐led decisions on the content and number of home visits offered by the family's PHN from pregnancy week 28 until the child is 2 years.

**Design:**

An interpretive description approach.

**Methods:**

Altogether 206 anonymized, undated reflection notes by PHNs becoming familiar with the programme were collected in 2017–2020. NVivo 12 and inductive content analysis were used to convert the data into manageable segments.

**Results:**

Focusing on childhood experiences and parental role expectations among prospective parents during home visits was seen as a major shift in the nurses' counselling strategy. Providing relevant information to the parents‐to‐be ahead of a meeting was important. Given sufficient staff and guidance, the programme was considered a good basis for building a relationship with the family.

## 1 INTRODUCTION

Becoming a mother is a vulnerable period and encompasses a process of developing competence (Hjälmhult & Lomborg, 2012). Nurses and midwives should be aware of the importance of postnatal social support for first‐time mothers, whose partners and mothers have been found to be key providers of such support (Leahy‐Warren et al., 2012). In a study aimed at discovering and comprehending conditions among parents during the transition to parenthood, the importance of having personal interaction with a trusted health professional was identified, to provide counselling and recognize feelings (Barimani et al., 2017). In Norway, there are well‐established maternity health care facilities connected to child health centres (Norwegian Directorate of Health, 2019). In 2019, 95% of pregnant women and their partners used this health care service (Statistics Norway, 2020). The municipal child health centres provide counselling to parents and their newborn child on a first home visit. After this, there are 13 regular encounters for the family and their under school‐aged child, i.e., 0–5 years of age, at the child health centre with a public health nurse (PHN). The universal and freely available programme of child health centres is based on health promotion and ill‐health prevention (Norwegian Directorate of Health, 2020). Although the programme is optional, the centres have a loyal following. In 2021, 78.1% of newborns received a home visit by a PHN and 93% of children aged 4 years underwent a health examination by a general practitioner through the child health centre. Further, within 3 days after homecoming with a newborn child, 53.2% of the families received a home visit from a municipal midwife (Statistics Norway, 2022). Central topics during the encounters at the child health centre include child development, breastfeeding, nutrition, vaccination and the parental role. The service providers at the child health centre are usually a PHN and a general practitioner, as well as a midwife and the general practitioner during the pregnancy (Norwegian Directorate of Health, 2019, 2020). Frequent home visits from a PHN, as regulated in the Norwegian child health centre programme and adapted to the needs of the mother postpartum, might have a preventive or therapeutic effect on postpartum depression symptoms (Glavin et al., 2009, 2010). A recent interview study among immigrant mothers in Northern Norway revealed that the experience of support, recognition and respect from the PHN was important for building a relationship based on trust between the mother and the PHN (Jensen & Clancy, 2021).

## 2 BACKGROUND

### 2.1 The New Families programme

In 2016, the city council of Oslo decided to provide the New Families programme (NF programme) as a supplement to the regular national child health centre programme (City of Oslo, 2021; Leirbakk et al., 2019). In the NF programme, PHNs offer home visits from week 28 of the pregnancy until the child is 2 years old. The targeted groups are couples or singles expecting their first child, or expecting their first child together, or expecting their first child in Norway (City of Oslo, 2021). If families are identified at the child health centre as having challenges in bringing up older children, these families are invited into the programme as well (Leirbakk et al., 2018, 2019). Relevant challenges could be identified by the PHN through her contact with the family or reported by a midwife, a general practitioner, or the child welfare service (City of Oslo, 2021). The NF programme, which is based on home visits to each family from their individual PHN, was implemented in all 15 districts in the City of Oslo in 2019. The programme is based on *proportionate universalism* as described by the Marmot Review Team (2010). According to proportionate universalism, actions must be universal and not targeted. However, their scale of intensity should be proportionate to the level of disadvantage (Carey et al., 2015). The NF programme is universal for its target groups and it emphasizes targeted services at the child health centre based on different needs among the families. The main goal is to ensure optimal development and growth in children and to diminish the need for expensive child protection services that might be ineffective (Leirbakk et al., 2018, 2019).

Previous studies have indicated that relational continuity of care has a positive impact on parents' evaluations regarding several aspects of maternity and child health centre services (Tuominen et al., 2014). Hence, a core idea in the NF programme is that the same PHN should follow the family throughout the consultations in the programme as well as in the regular child health centre consultations. The aim is to strengthen the relationship between the families and their PHN (City of Oslo, 2021). Because the programme is optional, based on proportionate universalism, and targets user‐led decisions on the focus and frequency of the home visits, it is designed to prevent stigmatization of families who might need home visits the most (City of Oslo, 2019).

To arrange the first home visit in the NF programme, the pregnant woman is usually informed by her midwife that the PHN will contact her by text message or telephone (City of Oslo, 2021). This first home visit paves the way for building a good relationship based on trust between the PHN and the prospective parent or parents. The user's manual for this programme includes examples of relevant conversation themes in the first meeting, such as the childhood history experienced by the parents‐to‐be, their expectations of their forthcoming role as parents, and hopes regarding their expected child. Applicable themes are also included in the manual for follow‐up in the subsequent meetings, for instance, about the parents' social life after the birth and the child's start at kindergarten (City of Oslo, 2021). A similar universally offered service based on tailoring the service to meet the needs of the family was described as likely to be effective by Dahl et al. (2014). A systematic review and meta‐analysis by Kendrick et al. (2000) indicated that home‐based interventions were associated with an improvement in the quality of the home environment.

During the implementation of the current NF programme in the City of Oslo, expert peers guided the PHNs during their first two NF home visits. The PHNs were asked to write reflection notes three times (every second month) about their own practice, working style and development (City of Oslo, 2021) (Table 1). These notes were discussed in professional meetings as a way to ensure that the PHNs understood the theory and methods underpinning this new programme and used them optimally according to the user's manual by City of Oslo (2021). Our study aimed to gain insight in benefits and challenges of the NF programme based on the PHNs' reflection notes. The research question was as follows: What reflections do PHNs have when implementing the NF programme?

**TABLE 1:** The template in the New Families programme for public health nurses' (PHNs') reflection notes.

A.	Describe a situation when you experienced that a home visit worked very well. What do you think made the visit work well?
B.	Describe a situation when you experienced a home visit as difficult for you. Do you have any thoughts about reasons for this? If you had a chance to implement the visit once more, what would you have done differently?
C.	What is important for you to emphasize in your further work with the New Families programme? How are you going to facilitate that?

*Note*: As part of their training, every second month the PHNs should set aside time for reflection and a short answer under A, B and C (three times altogether).

## 3 METHODS AND MATERIALS

### 3.1 Design

The inductive methodological orientation of interpretive description, described by Thorne (2016) was chosen because it makes it possible to mimic the interpretive mental attitude that characterizes the reasoning process of applied practice disciplines, like public health nursing. To organize and structure the data, this study used computer‐assisted qualitative data analyses produced using NVivo12 software (NVivo, n.d.). Interpretive description targets new knowledge in a form that appears meaningful and relevant to our current context and one may draw inspiration from specific methodological techniques (Thorne, 2016). In our study, the analytic procedure to convert the extensive data into manageable segments was based on using inductive content analysis, described by Polit and Beck (2017) and Graneheim and Lundman (2004). We have fulfilled the Standards for Reporting Qualitative Research when conducting and reporting this study (O'Brien et al., 2014).

According to Thorne (2016), researchers are invited to think from their disciplinary core in interpretive description. Hence, the authors have drawn on their familiarity with public health nursing gained over many years as practising PHNs, educators and researchers.

### 3.2 Collected data

The data consisted of 206 anonymized, undated reflection notes from PHNs collected in 2017–2020 in city districts by a specialist consultant for the NF programme in the City of Oslo. The reflection notes represented 11 of 15 city districts in total. In 72 reflection notes, the PHN's name was included but anonymized before use in the study. Hence, the number of PHNs in the study is unknown. It is likely that each PHN wrote two or three reflection notes. Each reflection note usually comprised one or two A4 pages and most were anonymous. A specialist consultant for the NF programme in the City of Oslo had sent the reflection notes to the study authors by encrypted e‐mail. The template for the reflection notes is presented in Table 1.

### 3.3 Ethics approval and consent to participate

The collected data were anonymized and treated as confidential. The NF programme's specialist consultant emailed an information sheet about the study to the PHNs employed in the City of Oslo. Participation by the PHNs was voluntary and they could withdraw without giving a reason if the reflection note could be identified by name. The Norwegian centre for research data approved the study (reference number: 759532).

### 3.4 Analysis

All 206 reflection notes were exported to NVivo 12 (NVivo, n.d.). The two authors then read through them several times to get an idea of what they were about. Next, we gained insight into patterns of similarities and differences in the content of the reflections. During this process, each reflection note was read, sentence by sentence. The first author then picked out almost every sentence as ‘a meaning unit’, 669 in total. After observing interrelations between some of these meaning units, the first author organized these units, ending up with 61 condensed meaning units. NVivo 12 was then used to organize a ‘code book’ showing the 61 condensed meaning units with the corresponding meaning units. After reading this code book in collaboration, the authors uncovered six main categories of reflections with related 16 sub‐categories (Table 2). Supplementary information about the analysis is available from the corresponding author.

**TABLE 2:** Six main categories in relation to the sub‐categories.

Main categories	Sub‐categories
1. Building a relationship before the child is born	Great value in meeting a couple before the birth of their child
	Meeting a single mother^a^ before the birth of her child
2. Reflecting the role of the father^a^ in the meeting	The father^a^ is being observed in the meetings
	Expectations of two parents^a^ participating in the meeting
3. Perceiving the purpose of the programme	A big change in public health nurses' (PHNs) counselling strategy
	An incongruence between what the parents^a^ demand and the idea about the content of the first meeting during pregnancy
	Good meetings
	The New Families programme as a resource‐intensive measure
	Challenges because of a new way of counselling
4. Preparedness as a prerequisite for the meeting	The conversation flows naturally
5. Challenges in relation to the set schedule	Parental scepticism about the programme
	Concerns and measures because of the COVID‐19 pandemic
	Feeling awkward about the meeting
	Typical obstacles in the meeting
6. Skills to customize counselling and increased responsibility	Self‐assessment by the PHN
	Interdisciplinary collaboration and peer counselling

^a^ = ‘to‐be’.

## 4 RESULTS

The analysis revealed six main categories (Table 2). These will be presented with quotes to illustrate the findings.

### 4.1 Building a relationship before the child is born

PHNs saw it as a privilege that due to the NF programme, they were allowed to spend optimal time with parents‐to‐be, based on their needs. Based on the first meeting, a good relationship with the parents was often achieved. Some PHNs expressed that families who had received home visits usually required less contact with the PHN later.

I have found that the mothers who have received extra [New Families] visits have become more confident and managed to relax a little. After each home visit, I have received no calls and messages or fewer than before.

A PHN emphasized that this programme should be offered universally to all parents.

Furthermore, I think the programme should be extended to apply to everyone the child health centre is in contact with. Although the transition from zero to one child is one of the greatest upheavals we experience as humans, for some people it may be just as stressful to have a second or third child because children are different, with different needs and challenges.

A recurring opinion was that both parents should participate in the meeting to create the optimal setting for a qualitatively good dialogue.

To bring the couple into focus, I think that for all home visits to the families it's important that both parents are present… the mother and the father are together around the child, and the process of getting the family and parenting tasks to function together is important. The parents should have a sense of fellowship and shared responsibility. As preventive health professionals, we should signal this.

… I have been on a home visit where only the mother was home. This was a useful experience because I found that the conversation turned out differently ‐ more like an interview, and the conversation did not flow as smoothly as when both parents are home …

To initiate a dialogue, the PHN usually introduced topics in the first home visit about the expectations among the parents‐to‐be about their roles as a father or a mother, and what values from their own childhood they would like, or not, to pass on to their own child. Childbearing was not usually a topic although the first home visit took place during the pregnancy. After the birth, the baby was included and the focus was no longer on the parents' life experiences.

It's very good to come home to a new family and actually have met the parents before, then it becomes a completely different meeting, we've come a long way already, and we can talk about important things, not only the presentation.

The focus of the relationship to a single mother was sometimes connected to reflections on her physical environment.

[single] mother seems conscientious and organized, […] the apartment seems newly refurbished, which the mother confirms. She wants orderliness around her.

### 4.2 Reflecting on the role of the father‐to‐be

A central topic in several reflection notes was that the PHNs had observed and assessed the father‐to‐be in his role and his contributions in the meeting. Mothers‐to‐be expressed their regret if their partner for some reason did not attend the meeting.

A focus area concerned what the fathers‐to‐be asked about and their responses to the PHN's counselling. For instance, some PHNs expressed doubt about whether some fathers‐to‐be really understood the idea of the home visit by the PHN and whether they were aware of they were expected to contribute to the dialogue. Most fathers‐to‐be met the expectations of being as active and participating as the mother‐to‐be in the conversations during the home visit before the child's birth.

The father says that they are, after all, a reasonably mature couple before having children, many of their friends are already past the baby stage, and they have received a lot of good advice and tips. They are concerned about reading up on scientific literature about infants to get more information, the father says they are keen to get this right.

Some of the fathers‐to‐be seemed withdrawn and quiet, not answering the PHN's questions in the encounters. Others signalled busyness, repeatedly looking at their wristwatch during the home visit.

The father came […] home late from work. In addition, he was also going back to work afterwards. I started noticing that the father kept looking at his watch during the conversation. Both the mother and the father responded to the topics that came up, but I gradually got the feeling that the father would rather have been somewhere else, and that he was ‘somewhat finished’ with the conversation. I ended up with a slight feeling that he didn't fully understand the purpose of all the questions.

### 4.3 Perceiving the purpose of the programme

The transition to conducting home visits before the child was born was often perceived as an immense change in the focus of the PHNs. Some of them described how they experienced difficulties in explaining this new programme to the families. A concern was how much questioning on parental background was acceptable and necessary for mutual relationship building with the parents.

There was sometimes an incongruence between the desire for information among the parents‐to‐be and what the PHNs had envisaged for the content of this first meeting. According to the user's manual, the first meeting before the birth should not focus on taking care of an infant and the practical concerns involved. Too much focus on this in the meeting might reduce the time available to focus on the previous life experiences and role expectations of the parents‐to‐be. However, some PHNs reflected on the importance of meeting the parents‐to‐be and fulfilling their needs before the birth by answering and counselling based on whatever queries they might have on practical concerns.

In particular, if the parents‐to‐be had experienced serious life events, like foetal death in an earlier pregnancy or other traumatic incidents, they usually appreciated having their own PHN to relate to. In some home visits after the child was born, the PHNs‐gained impressions of the child–parent attachment and interaction.

They were relaxed and at ease in their own home. Both the child and the parents seemed to feel secure, and the child was calm and sought her parents when I came too close – until she also gradually ventured closer to me, while checking with her parents whether it was safe and OK. Touching to see and lovely to see the contact the parents had with their child in familiar and safe surroundings, which was not as easy to see at the child health centre … where both children and parents probably become more stressed by the situation.

The NF programme was seen as resource‐intensive because of the extended time allocated for the home visits. PHNs mentioned need for enough staff and dedicated managers who approved the efforts involved in this new programme.

Now we have been working with New Families for almost a year. Something I constantly reflect on is that many need much closer follow‐up than the ordinary child health centre programme. Extra funding is supposed to be allocated for this, but in the last six months there has been a lot of sick leave at the child health centre and then there's less time anyway due to the lack of temporary staffing arrangements – and the regular consultations must also take place.

Some PHNs experienced that the guidelines were vague about documenting potentially demanding life situations, such as parental conflicts, in the parents' health records.

Counselling about breastfeeding was usually an example of a topic when the new programme was particularly relevant because the home visit facilitated the dialogue. In some reflection notes, PHNs maintained that access to sufficient competent personnel at the child health centre would eliminate the need for the NF programme as a supplement to the regular child health centre programme.

### 4.4 Preparedness as a prerequisite for the meeting

It was crucial for the PHNs to arrive well prepared, with an agenda for the home visit. Some PHNs pointed out that it might be inefficient use of time to conduct home visits to families who were not informed in advance about the aims and intentions of the NF programme.

For me, it is unusual that there was no baby there [at the home visit] that one could ‘focus on’. I also had an impression that the parents did not know exactly what the meeting was going to be about and were therefore a bit sceptical.

In particular, PHNs reflected on the importance of providing relevant information about the NF programme to immigrant parents. Good information to the parents‐to‐be could give them the confidence to decide what personal information they wanted to share in the meeting.

### 4.5 Challenges in relation to the set schedule

Although parents expressed a positive attitude to the programme, some of them did not agree to receive home visits. For instance, following the scheduled home visits was not possible if the parents expressed that they felt awkward because they experienced that their home was under observation during a home visit.

… certain mothers who don't fully understand the concept [of New Families]. They wonder what the purpose of the home visit is, and maybe they feel that they are being monitored …

Although some families expressed that they did not want the PHN's home visits, they often wished frequent contact with the PHN at the child health centre. Thus, it was emphasized that providing the home visiting programme might lower the general threshold among families for contacting the child health centre as well.

The COVID‐19 pandemic was an obstacle to conducting home visits during lockdowns. In the reflection notes, counselling by telephone or using video platforms such as Teams or Zoom was reflected on.

We still can't go on home visits because of the Covid pandemic, but video consultation is a good substitute.

A telephone call might not work well as a substitute for a home visit.

I've had another phone conversation, where I don't think it went that smoothly. I'm a person who looks closely at the face and body language of the people I talk to, so I missed that a lot.

If parents were working during the day, it was often not possible to find time for a home visit until the mother‐to‐be had started her maternity leave. This was usually 3 weeks before the expected date of delivery. In home visits this late, it was sometimes challenging to keep the attention on subjects other than preparations for the imminent birth.

### 4.6 Skills to customize counselling and increased responsibility

PHNs reflected on what they were used to, their own competences and perceived shortcomings in relation to the NF programme. The reflection notes thus highlighted the importance of getting guidance from skilled peers when starting to implement this new programme.

It was good to have a public health nurse educator with me the first time. We took turns speaking and both tried to follow the couple's narrative. I thought the ‘role model’ presented various topics in a good, natural way before she turned to the parents; I will take this with me.

A common topic in the reflection notes was a wish among the PHNs to continue the training of conversational skills to facilitate good conversations with the parents. Attending a Motivational Interviewing (MI) course was often part of the training related to the NF programme.

I want to keep practising […] specific and direct questions. […] [to continue] training in motivational interviewing

Because the PHNs often knew the families well due to their frequent and often close contact based on the NF programme, a typical reflection was that they felt a growing responsibility for the families. To cope with this, it was important to receive professional guidance regularly.

As a public health nurse, I feel that I have got to know the families, and perhaps that I have greater influence in the families I have visited. When I am more accessible, I am asked more, and my responses/considerations are followed to a greater extent, I think. Which is fine, but on the other hand results in more responsibility.

To provide relevant counselling to the families, interdisciplinary collaboration was seen as pivotal. The midwife, the general practitioner and the psychologist connected to the current child health centre were mentioned as customary and relevant partners in this context. Sometimes, referrals were made to the child protection service for collaboration.

## 5 DISCUSSION

In the reflection notes, the NF programme was primarily described as an advantage because it facilitated good relationships with the families prenatally. Accordingly, it was recommended that it should be offered universally, not only to targeted groups like first‐time parents. According to Ketner et al. (2019), having several children is typically connected to parents being better at providing basic care for their child than first‐time parents are. At the same time, first‐time parents are not necessarily more vulnerable than parents with older children. For instance, parents with more than one child might be disadvantaged in terms of relationship quality and well‐being (Ketner et al., 2019). Thus, they might benefit from participating in the NF programme. Moreover, offering the programme to all parents and at the child health centre might contribute to reducing social stigmatization as described by Frost (2011), of those who needed support the most. Universal provision of the programme might also prevent possible health inequalities among families. This is supported by the theory of proportionate universalism. That is, providing the interventions solely to the people most in need will not reduce health inequalities sufficiently (Marmot, 2010). Because counselling should be proportionate to the family's needs, one should not confuse the ‘impartiality’ of counselling based on universalist policies with ‘sameness’ of counselling (Carey et al., 2015). In this respect, the NF programme and its efforts to provide home visits to parents‐to‐be might thus support PHNs in adapting their counselling to the needs of any family, because they have built a relationship prenatally. This might hinder sameness of counselling later.

The reflection notes highlighted how the home visiting programme provided an opportunity to focus on the life experience of the parents‐to‐be and their transition to parenthood prenatally. Once the child was born, it was no longer perceived as appropriate to focus on these parental transition perspectives in the encounters between the PHN and the first‐time parents with a new‐born child. Similar reflections of tensions regarding a shared focus on the baby and the mother's wellbeing during postnatal home visits by family health nurses were seen in a study by Shepherd (2011).

When providing the home visits prenatally, the PHNs emphasized that it was important that the parents should be well informed in advance about the NF programme. It was especially important to provide relevant information before a home visit to immigrant parents. This is supported by the findings in a recent study by Jensen and Clancy (2021) among immigrant mothers of new‐born children in Northern Norway. These mothers had received limited information about the purpose and content of the PHN's home visits. For instance, they did not know that these visits were optional (Jensen & Clancy, 2021). Another study indicated that few women preferred a prenatal home visit from the PHN because they had an inadequate understanding of the purpose of this prenatal contact (Olander et al., 2019).

The NF programme promoted an open dialogue with the parents‐to‐be about topics that were closely related to their personal experiences and transition to parenthood. Some PHNs saw this as a new focus and thus challenging. The PHNs' own conversation skills and the usefulness of MI, particularly in relation to its open‐ended questions and reflective listening as presented by Beckwith and Beckwith (2020), were highlighted in the reflection notes. Some PHNs described a desire to improve their conversation skills to enable better communication in open discussions with the users.

According to the reflection notes, the mentoring by expert peers was rather informal and was appreciated because it helped the PHNs to gain the knowledge, skills, and confidence needed when counselling the prospective parents. This mentoring was thus experienced as a deliberating and intentional activity as described by Shellenbarger and Robb (2016).

The PHNs' observations of the role of the father‐to‐be and his participation in the meeting was a recurring theme in the reflection notes. In some situations, they saw how the father‐to‐be had a central role in facilitating a good dialogue in the meeting. In other situations, his role did not function as well, maybe because he seemed not to be sure about what was expected of him in a home visit. This corresponds to the results in a recent interview study by Solberg et al. (2021). Another study revealed that fathers usually had few expectations for the PHNs' home visits. Moreover, after the home visits they often felt disappointed and left alone (Solberg & Glavin, 2018).

An exploratory survey among mothers and fathers by Ketner et al. (2019) indicated that interventions focusing on relationship quality and parenting behaviour could be effective because they increased parents' well‐being and facilitated their own transition to parenthood (Ketner et al., 2019). However, in the NF programme there was no focus on the parents' mutual relationship quality as a couple during the prenatal visits. Some reflection notes suggested that there was lack of focus on the couples' relationship during their transition to parenthood. The relationship of the couple and related follow‐up questions are presented as a topic for later home visits after the child's birth (City of Oslo, 2021). Further research should assess the impact of the NF programme on population level and integrate a health economic evaluation, as described by the Norwegian Institute of Public Health (2022).

### 5.1 Strengths and limitations of this study

Transparency has been central in all steps of conducting the study, as described by Polit and Beck (2017). Further, the study took advantage of the rich data available. The computer software NVivo12 (NVivo, n.d.) played a key role in structuring the data. We had no demographic data about the PHNs or their employment or on which of the reflection notes were written by the same PHN. This is a limitation, for instance, because we could not find out whether the reflections might have changed over time or whether the data reflected variation between child health centres and city districts. A further limitation was that because the data had already been collected, different nuances in the text could be hard to identify.

## 6 CONCLUSIONS

Based on the reflection notes, the NF programme was seen as a clearly defined extension of the regular child health centre programme. The PHNs emphasized that conducting a home visit to parents expecting their first child was usually seen as a contribution to a good relationship between the PHN and the family. Most of the parents‐to‐be seemed willing to receive these home visits. The two‐way dialogue between the PHN and the parents in this meeting had a new focus based on the parents' previous life experience, their wish to bring further elements based on their own positive and negative childhood experiences and their expectations for becoming parents very soon. This new practice was seen as an important contribution to the transition to parenthood. The PHNs assessed this programme as an advantage for performing their job in a satisfactory way, based on the family's needs and desires. Not all the parents saw the point of receiving a home visit. Regardless, this programme might help lower the threshold for families in general for taking more contact with the child health centre. The findings suggest that, from the PHN's perspective, integrating the NF programme at the child health centres mainly results in benefits. Integrating this new programme requires guidance in a couple of home visits prenatally from an experienced PHN colleague during the implementation phase.

## FUNDING INFORMATION

This research was in part funded from The Research Council of Norway – Project Number: 282167.

## CONFLICT OF INTEREST STATEMENT

No conflict of interest has been declared by the authors.

## ETHICS STATEMENT

The Norwegian centre for research data approved the study, reference number: 759532.

## Data Availability

Research data are not shared.
